# Using a multiplex serological assay to estimate time since SARS-CoV-2 infection and past clinical presentation in malagasy patients

**DOI:** 10.1016/j.heliyon.2023.e17264

**Published:** 2023-06-13

**Authors:** Mame Diarra Bousso Ndiaye, Lova Tsikiniaina Rasoloharimanana, Solohery Lalaina Razafimahatratra, Rila Ratovoson, Voahangy Rasolofo, Paulo Ranaivomanana, Laurent Raskine, Jonathan Hoffmann, Rindra Randremanana, Niaina Rakotosamimanana, Matthieu Schoenhals

**Affiliations:** a Institut Pasteur de Madagascar, Antananarivo, Madagascar; b Medical and Scientific Department, Fondation Mérieux, Lyon, France

**Keywords:** SARS-CoV-2 antibodies, COVID-19 seroprevalence, Prediction model, Madagascar, Symptom presentation, Time since infection

## Abstract

**Background:**

The world is facing a 2019 coronavirus (COVID-19) pandemic caused by severe acute respiratory syndrome coronavirus 2 (SARS-CoV-2). In this context, efficient serological assays are needed to accurately describe the humoral responses against the virus. These tools could potentially provide temporal and clinical characteristics and are thus paramount in developing-countries lacking sufficient ongoing COVID-19 epidemic descriptions.

**Methods:**

We developed and validated a Luminex xMAP® multiplex serological assay targeting specific IgM and IgG antibodies against the SARS-CoV-2 Spike subunit 1 (S1), Spike subunit 2 (S2), Spike Receptor Binding Domain (RBD) and the Nucleocapsid protein (N). Blood samples collected periodically for 12 months from 43 patients diagnosed with COVID-19 in Madagascar were tested for these antibodies. A random forest algorithm was used to build a predictive model of time since infection and symptom presentation.

**Findings:**

The performance of the multiplex serological assay was evaluated for the detection of SARS-CoV-2 *anti*-IgG and *anti*-IgM antibodies. Both sensitivity and specificity were equal to 100% (89.85–100) for S1, RBD and N (S2 had a lower specificity = 95%) for IgG at day 14 after enrolment. This multiplex assay compared with two commercialized ELISA kits, showed a higher sensitivity. Principal Component Analysis was performed on serologic data to group patients according to time of sample collection and clinical presentations. The random forest algorithm built by this approach predicted symptom presentation and time since infection with an accuracy of 87.1% (95% CI = 70.17–96.37, *p-value* = 0.0016), and 80% (95% CI = 61.43–92.29, *p-value* = 0.0001) respectively.

**Interpretation:**

This study demonstrates that the statistical model predicts time since infection and previous symptom presentation using IgM and IgG response to SARS-CoV2. This tool may be useful for global surveillance, discriminating recent- and past- SARS-CoV-2 infection, and assessing disease severity.

**Fundings:**

This study was funded by the French Ministry for Europe and Foreign Affairs through the REPAIR COVID-19-Africa project coordinated by the Pasteur International Network association. WANTAI reagents were provided by WHO AFRO as part of a Sero-epidemiological “Unity” Study Grant/Award Number: 2020/1,019,828–0 P·O 202546047 and Initiative 5% grant n°AP-5PC–2018–03-RO.

## 1 Introduction

Coronavirus disease 2019 (COVID-19) is caused by severe acute respiratory syndrome coronavirus 2 (SARS-CoV-2) [1]. As of the April 8, 2022 the WHO has reported 494 million confirmed cases and more than 6 million deaths worldwide from this disease [2]. Most people infected with SARS-CoV-2 have mild to moderate respiratory illness and recover without the need for specific treatment [3]. However, the elderly and those with underlying medical conditions such as cardiovascular disease, diabetes, chronic respiratory disease and cancer are more likely to develop severe presentations.

PCR-based tests are widely used to diagnose active infection to SARS-CoV-2 [[4], [5], [6], [7]]. The two most common target genes are open reading frame 1 ab (ORF1ab) and nucleocapsid protein (N). The SARS-CoV-2 genome encodes 20 proteins including 16 non-structural and 4 structural proteins. The infected patient's immune system will produce antibodies against all of these viral proteins in the serum. Most SARS-CoV-2 developed serological tests target antibodies against spike glycoprotein (S), or nucleocapsid protein (N) antigen because of the high antigenicity of those proteins. The S protein consists of two subunits: S1 and S2 [8,9]. S1 allows the binding and entry into target cells. Through its receptor binding domain (RBD), S1 interacts with the human angiotensin-converting enzyme 2 (ACE2) receptor. The RBD of the S1 subunit is the primary target of neutralizing antibodies [10]. N plays the main role in the genomic replication, transcription and packaging of the virus [11].

Antibodies to SARS-CoV-2 are produced a few days to weeks after viral infection [12]. The presence of antibodies indicates that a person has been infected with the COVID-19 virus, whether the individual has severe or mild disease, or even asymptomatic infection.

Quantification of antibody levels can be very informative. It is an important indicator associated with the duration of the disease, time since infection, severity of symptoms. Moreover, in the case of COVID-19 infection, patients with mild or moderate disease may also experience prolonged symptoms presentation, known as Long COVID [13].

IgM antibodies are produced during the early stages of infection, whereas IgG antibodies, which have a higher target protein affinity, are markers of the immune response developed later after infection but persist over time and provide longer protection against the antigen. In the humoral response against SARS-CoV-2, IgG or IgM appears to increase within 20 days of symptom onset [[14], [15], [16]].

Serological tests are commonly used to detect a virus circulation in the population and provide an indication about the proportion of this population that may be immunized against the virus. Since the beginning of the pandemic, more than 200 serological tests dedicated to detect SARS-CoV-2 have been developed [17].

A better characterization of the humoral response to SARS-CoV-2 infection would be of great value in estimating the time of infection, or retrospectively the patient's clinical presentation. Studies have shown that by measuring antibodies in serum samples from infected patients, it is possible to estimate the time since infection and to assess the serologic reconstruction of past SARS-CoV-2 transmission [18].

Serological diagnostic tests generally classify a sample as positive if the measured antibody level is above a defined threshold. In this study, we developed and validated a multiplex serological tool based on the Luminex xMAP® technology, evaluated its performance against 4 antigens (S1, S2, RBD and N) for the detection of SARS-CoV-2 specific IgM and IgG antibodies in a cohort of COVID-19 confirmed patients. Indeed, the serological responses are context specific and may be influenced by the circulation of multiple coronaviruses strains, genetic background and endemic pathogen circulation [19,20]. The serological data collected were then used to estimate the time since infection and to retrospectively describe symptomatic presentations.

## 2 Materials and methods

### 2.1 Development of a luminex xMAP® multiplex assay

- Bead coupling

The first step in the development of the assay was the coupling of the beads with the SARS-CoV-2 S^1^, S2, RBD and N antigens. Coupling of the proteins onto the microspheres was performed according to the manufacturer's instructions [21]. The carboxylated magnetic beads (MagPlex™) and the coupling kit (Luminex, 40–50016) were supplied by Luminex Corporation (Austin, TX, USA). The recombinant proteins used are listed in the Supplementary Table 3. Briefly, 5.10^6^ microspheres were transferred to low-binding tubes, positioned into Dynamag-spin magnet (Invitrogen, 12320D), and resuspended in 500 μL activation buffer (0.1 mol/L sodium phosphate, pH 6.2). Then, 10 μL of Sulfo-NHS (50 g/L) and 10 μL EDC (50 g/L) were added and the suspension was incubated for 20 min in the dark at room temperature (RT). The activated microspheres were washed twice in the activation buffer. 5 μg of recombinant protein for 10^6^ beads (diluted in activation buffer to a total of 500 mL), were added and the mixture was spun down for 2 h in the dark. After incubation, the microspheres were three times washed in wash buffer. Finally, the beads were resuspended in 1 mL of wash buffer and stored at 4 °C in the dark. The coupled beads were counted with Malassez cells to adjust the concentration. The following bead regions were used: MC10012-01 (Spike S1), MC10013-01 (Spike S2), MC10014-01 (Spike RBD), MC10015-01 (Nucleocapsid).

For confirmation of coupling, a dilution range (0.625–4 μg/mL) of goat anti-human IgG Fc labelled with phycoerythrin detection antibody (Thermo Fisher Scientific, H10104) was tested with the antigen-coupled beads. The median fluorescence intensity (MFI) was detected by a standard PMT.

- Validation of the 4 Plex assay

The coupling was confirmed using a dilution of the detection antibodies (Supplementary Fig. 1A). MFI levels related to the antibodies concentration of 2 μg/mL was defined as the saturation concentration and was used for the downstream experiments.

The detection limit for antibodies quantification was then assessed. The linearity zone indicating the detection range was found between 0.1 ng/mL and 1000 ng/mL for all four antibodies tested (Supplementary Fig. 1B). The repeatability and the reproducibility of the assay were evaluated with the intra-assay variation (intra-assay CV) and the inter-assay variation (inter-assay CV) respectively (Supplementary Table 2).

Cross-reactivity of the multiplexed assay was evaluated by testing mixed sets of coupled beads, individual specific antibodies and individual detection antibodies (IgG or IgM) to determine any cross-reacting antigens with the non-targeted beads.

The lower limit of detection (LOD) of the test was defined as the mean blank MFI + 3 Standard deviation (SD). Lower limit of quantification (LLOQ) estimation was based on repeated sample measurements (n = 6) with 0.01, 0.1, or 1 ng/mL of antibody, whereas upper limit of quantification (ULOQ) was based on repeated sample measurements (n = 6) with 100, 1000, or 10,000 ng/mL. Inter-assay and intra-assay variations was assessed by analyzing multiple replicates (intra-assay n = 12, inter-assay n = 6) of a control sample with a known concentration for each protein (Supplementary Table 2).

### 2.2 Multiplex luminex assay

After verifying that the coefficient of variation (CV) for the number of beads counted in each region is less than 15%, all beads were mixed. The volume of mix to be dispensed was set to have 1000 beads/region/well. Each well received 100 μL of positive control, plasma (diluted to 1:100) and assay buffer only (blanks). Round-bottom polystyrene 96-well microplates (Costar, CORNING_ 3915) were used. The plate was incubated for 30 min on a plate shaker, then placed 60s on a plate magnet to pull down magnetic microspheres and washed with assay buffer. This washing step was repeated twice. Detection antibodies (4 μg/mL) were added to each well and incubated for 30 min (100 μL/well). Microspheres were then washed twice and resuspended in 120 μL of assay buffer before analysis on the MagPix™ instrument supplied by Luminex Corporation (Austin, TX, USA). All incubations were performed in the dark, at room temperature, on a plate shaker (900 rpm).

The fluorescence background was determined by the mean of MFI +3 SD. The MFI shown in the figures is the median fluorescence minus the fluorescence background. Cut-off limits for the determination of positive antibodies in the SARS-CoV-2 infected individuals were determined by receiver operating characteristics (ROC) analysis.

### 2.3 Comparison with the ELISA kits

The performance of the Luminex test was compared to two commercial ELISA kits: ID Screen® SARS-CoV-2-N IgG Indirect ELISA (SARSCoV2S–8 P, ID. Vet, Grabels, France) which detects antibodies (IgG) directed against the nucleocapsid (N) of the SARS -CoV-2 in human serum or plasma, and the WANTAI SARS-CoV-2 Ab ELISA (WS-1096, Beijing Wantai Biological Pharmacy Enterprise Co., Ltd.) which determines IgA, IgG, IgM antibodies to the SARS-CoV-2 Spike RBD antigen.

### 2.4 Study population and sample collection

The FFX (first few cases) cohort from the Institut Pasteur of Madagascar was described in a previously study [22]. Among the FFX cohort, 43 Malagasy patients with positive SARS-CoV-2 PCR tests were included in the present study. These patients were enrolled from 3 different Hospitals of Antananarivo Madagascar: They were followed at 7-day intervals, at least until day 21, or longer until their PCR was negative. Patients were followed-up at home for one year (at months 1, 3, 6, and 12 of their SARS-CoV-2 test diagnosis). Of the 43, only 13 completed the 12-month visits. All participants were screened for comorbidities. Informed consent forms were obtained from all patients prior to enrolment in the study, in accordance with the FFX core protocol. All patients’ characteristics are summarized in Supplementary Table 1. This study protocol obtained ethical approval from the Biomedical Research Ethics Committee of Madagascar (n°. 058/MSAN/SG/AGMED/CERBM, March 30, 2020 and amendment n°109/MSAN/SG/AGMED/CERBM, June 24, 2020). As negative control, 40 pre-epidemic sera collected in 2015 from Malagasy individuals were tested. No individuals in this study were vaccinated against COVID-19 as individuals were recruited and followed-up in 2020 prior to the vaccination campaign in Madagascar.

Participants had peripheral blood samples collected during follow-up at each timepoint. Blood was collected in two 5 mL heparin tubes. Serum was separated from the red fraction of blood (red and white blood cells) and stored at −80 °C.

### 2.5 Statistical analysis

Luminex data were analyzed using the Prism version 9 software (GraphPad, La Jolla, CA). The Mann Whitney test was used to compare differences in MFI between symptomatic and non-symptomatic participants and to compare positive and negative samples for *anti*-SARS-CoV-2 antibodies. ROC curves were generated to determine the detection thresholds, sensitivity and specificity values. We compared PCA scores by performing Wilcoxon signed rank exact test and Mann-Whitney U tests.

We performed a Random Forest analysis and a PCA analysis for prediction and estimation of time to infection and symptom presentation. The random forest analysis was performed using a validation set approach, which involves randomly dividing the data into two sets: one set is used to train the model and the other set is used to test the model. In our case, 80% of the data set was used for training a linear regression model and 20% was used to evaluate the performance of the model. This set of analyses was performed on R using the packages random Forest version 4.6–14 and FactoMineR version 2.4 and ggplot for PCA. The R-Scripts used for this study are available in the Statistical tools for high-throughput data analysis (STHDA) website [23].

## 3 Results

### 3.1 Performance of the luminex xMAP® assay for the serological detection of SARS-CoV-2

**Fig. 1:**
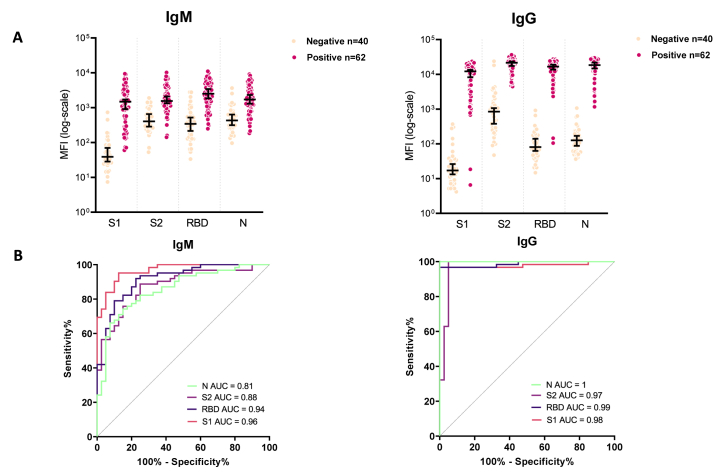
**Performance of the luminex 4-plex assay.** (A) Boxplot representing MFI levels for of S1. S2. RBD. N between negative samples (40) and positive samples (N = 34) IgM and IgG. Negative samples are pre-endemic sera collected in 2015. Positive samples are collected from hospitalized patients with a positive PCR for SARS-Cov-2. (B) Receiving Operating Characteristic (ROC) curve obtained with MFI of negative and positive. MFI: Median Fluorescence Intensity.

We investigated the levels of *anti*-IgM and *anti*-IgG targeting S1, S2, RBD and N between the pre-epidemic negative control (n = 40) and positive patients at different timepoints after a positive PCR test (n = 62) from the FFX cohort described in the methods section, based on the MFI generated by the Luminex test (Supplementary Table 1). For each combined target, MFI levels in positive patients were significantly higher than in negative controls (Fig. 1A and B). Moreover, we observed that the MFI intensity was 4-fold higher for IgGs (median MFI = 10,771) than for IgMs (median MFI = 1280).

Two pre-epidemic negative samples strongly expressed *anti*-S2 IgGs (MFI >10,000). To ensure that this was a coronavirus non-specific signal, we tested these samples for *anti*-S2 IgGs from seasonal human coronaviruses OC43, NL63, 229 E and HKU1 and found positivity for *anti*-S2 IgGs from OC43 and HKU1 in one sample and positivity for *anti*-S2 IgGs from NL63 in the other sample, suggesting cross reactivity between the SARS-CoV-2 S^2^ antigen subunit and antibodies developed against seasonal human coronaviruses Spike proteins (Supplementary Fig. 2).

Sensitivity (se), specificity (sp), and threshold values were obtained using ROC curves. For IgM, specificity reached 95% for all of the antigens. The S1 had better performance for IgM (AUC = 0.96; *p-value* <0.0001). In contrast, N had the lowest diagnostic score among the four antibodies (AUC = 0.81; *p-value* <0.0001) (Table 1 and Fig. 1B). For IgG antibody detection, all targets had sensitivity and specificity values > 95%, unlike IgM antibodies (Table 1 and Fig. 1B). The threshold of protein detection was set with the highest possible specificity value.

**Table 1:** **Performance of the 4-plex assay.** Sensitivity and specificity of the luminex antibody assay for antigen in multiplex.

		AUC	95% CI	p value	cut off	Sensitivity %	95% CI	Specificity %	95% CI
IgM	**S1**	0.96	0.94–0.99	<0.0001	>201.90	83.87	72.79–91.00	95.00	83.50–99.11
	**S2**	0.88	0.81–0.94	<0.0001	>1437	56.45	44.09–68.06	97.50	87.12–99.87
	**RBD**	0.94	0.89–0.99	<0.0001	>2036	62.90	50.46–73.84	95.00	83.50–99.11
	**N**	0.81	0.71–0.91	<0.0001	>1404	58.06	45.676–9.52	95.00	83.50–99.11
IgG	**S1**	0.98	0.95–1.00	<0.0001	>335.70	96.77	88.98–99.43	97.50	87.12–99.87
	**S2**	0.97	0.94–1.00	<0.0001	>4396	100.00	94.17–100.00	95.00	83.50–99.11
	**RBD**	0.99	0.97–1.00	<0.0001	>1613	96.77	88.98–99.43	100.00	91.24–100.00
	**N**	1.00	1.00–1.00	<0.0001	>1114	100.00	94.17–100.00	100.00	91.24–100.00

Two hundred and fifty eight samples from 43 patients collected between day 1 and day 180 were tested with two commercially available kits. 16.27% of samples were negative for SARS-CoV-2 antibodies (n = 42) with the Wantai kit and 30.23% with the ID. Vet kit (n = 78). Sera from these negative patients were then tested for SARS-CoV-2 IgM and IgG antibodies using the Luminex xMAP® assay. Of the negative sera tested with the ID. Vet kit, 39.74% (31/78) were positive for IgG N antibodies. For the negative Wantai, 54.76% (23/42) were positive for *anti*-RBD IgM and IgG antibodies (Supplementary Fig. 3). The developed Luminex xMAP® assay is able to detect more seropositivity compared to the ELISAs assay regarding the sensitivity of the multiplex test.

### 3.2 COVID-19 symptomatic individuals produce more RBD-specific IgM

**Fig. 2:**
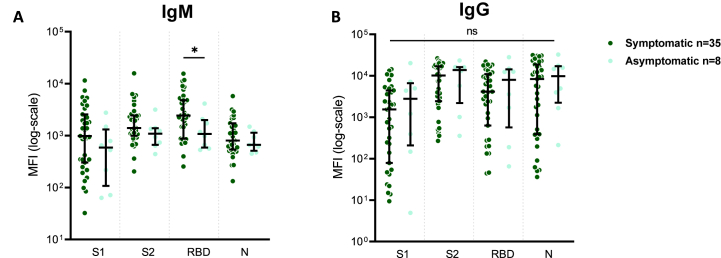
**Boxplot representing MFI levels for of S1. S2. RBD. N** between symptomatic (n = 35. Dark green) and non symptomatic patients (N = 8. light green) IgM (A) and IgG (B). Error bars represent Median with interquartile range. MFI: Median Fluorescence Intensity. Data were compared using the Mann-Whitney test. MFI levels are represented in log-scale. *: *p-value* <0.05, ns: non significative

We investigated the difference in antibodies levels between symptomatic (n = 35) and asymptomatic COVID-19 patients (n = 8). No differences were found when *anti*-S1, S2, RBD, and N IgG were studied and only the MFI values associated with *anti*-RBD IgM were significantly higher in symptomatic patients compared to asymptomatic ones (*p-value* 0.0333) (Fig. 2). This trend was confirmed using commercial IDVET (*anti*-N IgG) and Wantai (*anti*-RBD IgM and IgG) kits but no significant difference was observed.

### 3.3 IgM and IgG responses to SARS-CoV-2 show distinct patterns over time

**Fig. 3:**
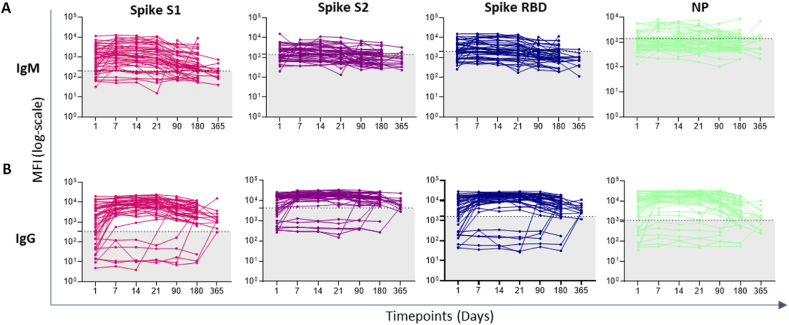
**Kinetics of seroconversion with 7 timepoints from day 1 to day 365.** (A) line plot of antibody detection in 43 hospitalized patients in IgM. (B) line plot of antibody detection in IgG. Each line represents a patient. Dashed line and grey area indicate the cut off of positivity. MFI: Median Fluorescence Intensity. MFI levels are represented in log-scale.

To assess IgM and IgG antibody seroconversion, survival curves for the four antigens were plotted. More than 60% of patients had IgG positive antibodies specific for S1, RBD, and N in the first 20 days before decreasing at 3 months (Fig. 3). For IgG, antibodies decreased by day 180, but the majority of patients remained positive at 1-year post-infection (day 365) (Fig. 3). For *anti*-S2 IgM, 79% (34/43) of patients had subthreshold MFI values at 6 months. At 1-year post-infection, seroconversion rates were 100%, 76%, 72%, and 71% for *anti*-S1, S2, RBD, and N IgM respectively.

For IgG antibody detection, we observed the same antibody seroconversion kinetics for all four antigens. 1/43 patients showed late IgG antibody conversion at day 21 and 2/43 showed seroconversion 3 months after the day of symptom onset (Fig. 3). After the first year of infection, the seroconversion rate was 95%, 100%, 100%, and 95% for *anti*-S1, S2, RBD, and N IgG, respectively.

**Fig. 4:**
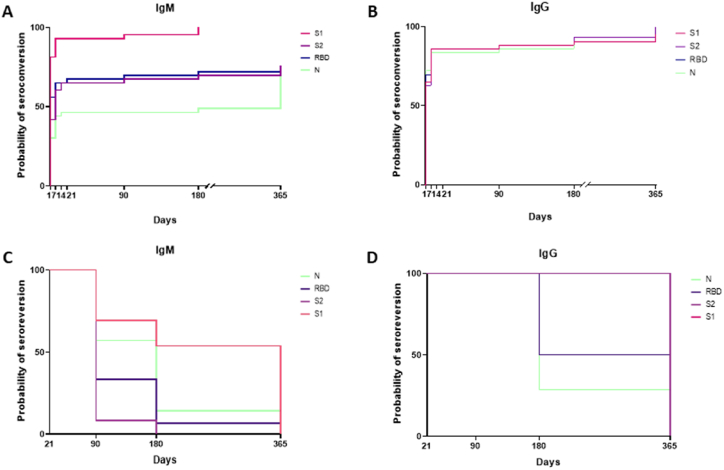
**Seroconversion and seroreversion in IgM and IgG antibodies over 365 days of follow-up.** A and B, Kaplan-Meier plots of patients with IgM and IgG seroconversion, respectively. C and D, Kaplan-Meier plots of patients with IgM and IgG seroreverted respectively.

Seroconversion curves showed that by day 1, more than 50% of all patients had seroconverted and showed *anti*-S1 IgG or IgM seropositivity. These patients reached more than 80% seroconversion at 6 months of follow-up (Fig. 4 A-D and Supplementary Table 4). The IgM antibody seroconversion rate at day 1 was 41.86%, 50%, and 30.23% for *anti*-S2, *anti*-RBD, and *anti*-N antibodies, respectively. At 6 months, these rates increased but did not exceed 55% for *anti*-N IgM (Supplementary Table 4).

**Fig. 5:**
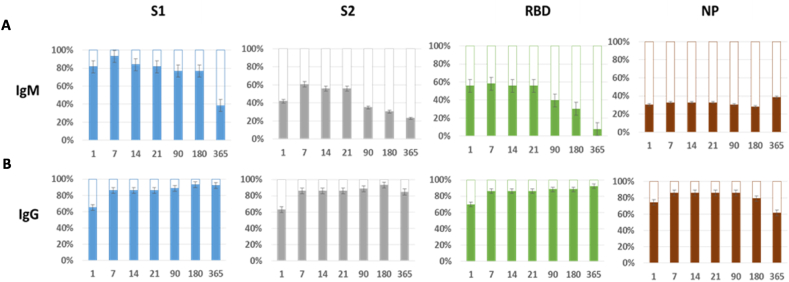
**Seroprevalence of IgM and IgG in the FFX cohort.** Antibody seroprevalence from day 1 to Month 12 (day 365) for *anti*-SARS-Cov-2 S^1^ (blue), S2 (grey), RBD (green) and N (red) with error bars of the 43 patients of the FFX cohort. A represent IgM data and B IgG data.

At day 7, over 90% of the cohort is positive for *anti*-S1 IgM. After 90 days of the infection, less than 40% of patients are positive for *anti*-RBD. For S2 and RBD, from day 1 to day 180, the positivity range is between 60% and 30%, before decreasing at 23% and 8% for *anti*-S2 and *anti*-RBD respectively. For *anti*-N IgM, the positivity range is between 40% and 20% during the 6 first months of infection and reached 40% at 12 months (Fig. 5).

For IgG detection, antibody positivity against all targets was greater than 60% on day 1 and increased during follow-up to more 80%, with the exception of *anti*-N, which decreased to 60% at 12 months (Fig. 5).

### 3.4 Time since infection and clinical presentation impact on serology results

The observed distinct antibody-dependent kinetics enabled us to investigate the possibility of estimating time since infection and, potentially, prior clinical presentation.

**Fig. 6:**
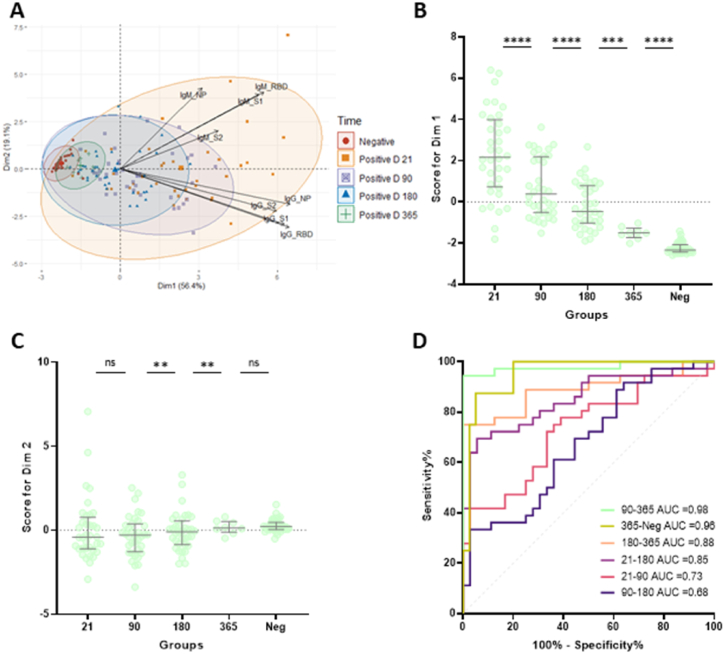
**PCA representing time since infection using serology results.** Principal component analysis (PCA) was performed on the expression of *anti*-S1, *anti*-S2, *anti*-RBD and *anti*-N IgM and IgG. A. Explanation of variance between early infections (day 21), 3-month infections (day 90), 6-month (day 180) and 1-year infections (day 365). N = 36 samples at each time point except 12 months (n = 8) and Negative (n = 40). Each point represents one patient. Color coding represents infection date groups. The axes represent principal components 1 (Dimension 1, Dim1) and 2 (Dimension 2, Dim2) and the percentages indicate their contribution to the total observed variance. Axis values represent individual PCA scores. The concentration ellipses correspond to 90% data coverage. Arrows represent the contribution of each IgG and IgM to the variance described by Dim1 and Dim2. B. Distribution of individual PCA score values as a function of infection date and time point, for Dim1. Data were compared using the Wilcoxon rank sum test. ****: *p-value* <0.0001. and Mann- Whitney test ****: *p-value* <0.0001 for comparisons between negative and 12 month. C. Distribution of individual PCA score values as a function of infection date and time point, for Dim2. Data were compared using the Wilcoxon rank sum test. ****: *p-value* <0.0001. and Mann- Whitney test ****: *p-value* <0.0001 for comparisons between negative and 12 month. D. Receiving Operating Characteristic (ROC) curve obtained PCA Scores for Dim1. Color coding represents ROC of comparison for different infection date groups

A principal component analysis (PCA) was performed using the combination of all variables in IgM and IgG (Fig. 6A–D). The PCA profiles were mostly separated when divided by infection date group (Fig. 6A). Dimension 1 explained 56.4% of the total observed variance, compared with 19.1% for Dimension 2 (Supplementary Fig. 4). The main variables explaining the variance described by Dimensions 1 and 2 were MFIs of RBD-targeting IgM and IgG, with the least contributing variable being MFIs of *anti*-S2 IgM. (Fig. 6A).

The coordinates on Dim 1 and Dim 2 of the PCA were used to describe a score for each individual (Fig. 6). Patients in the cohort were divided into 4 infection groups: 21 days, 90 days, 180 days, 365 days of infection and negative group. The Dim 1 score was highest at day 21 and decreased significantly over time (*p-value* <0.0001) (Fig. 6B). A ROC analysis was performed to characterize each group. In doing so, we found that samples collected at 3 months versus 1 year showed the best discrimination (AUC = 0.98, 95% CI = 0.94–1; Specificity = 100% and sensitivity = 94.44%) (Fig. 6D). We found good discriminations between samples collected at 6 months and 1 year (AUC = 0.89, 95% CI = 0.79–0.99; Specificity = 100% and sensitivity = 75%) (Fig. 6D and Table 2). Samples collected at 3- and 6-months post-infection, however, were difficult to distinguish, suggesting stable humoral profiles at these timepoints (AUC = 0.68, 95% CI = 0.55–0.80; Specificity = 97.22% and sensitivity = 33.33%) (Table 2).

**Table 2:** Discrimination of different time of infection groups using PCA dimensions as scores.

	AUC	95% CI	p value	cut off	Sensitivity %	95% CI	Specificity %	95% CI
<1 month–3 month	0.73	0.61–0.85	0.0008	>2.84	41.67	27.14–57.80	97.22	85.83–99.86
<1 month–6 month	**0.85**	**0.76–0.94**	**<0.0001**	**> 1.61**	**63.89**	**47.58**–**77.52**	**97.22**	**85.83**–**99.86**
3 month–6 month	0.68	0.55–0.80	0.0106	>1.76	33.33	20.21–49.67	97.22	85.83–99.86
3 month–12 month	**0.98**	**0.94–1.00**	**<0.0001**	**> -1.00**	**94.44**	**81.86**–**99.01**	**100.00**	67.56–100.00
6 month–12 month	**0.89**	0.79–0.99	0.0007	> −0.99	75.00	58.93–86.25	100.00	67.56–100.00
12 month - Negative	0.96	0.90–1.00	<0.0001	> −1.59	75.00	40.93–95.56	97.5	87.12–99.87

**Fig. 7:**
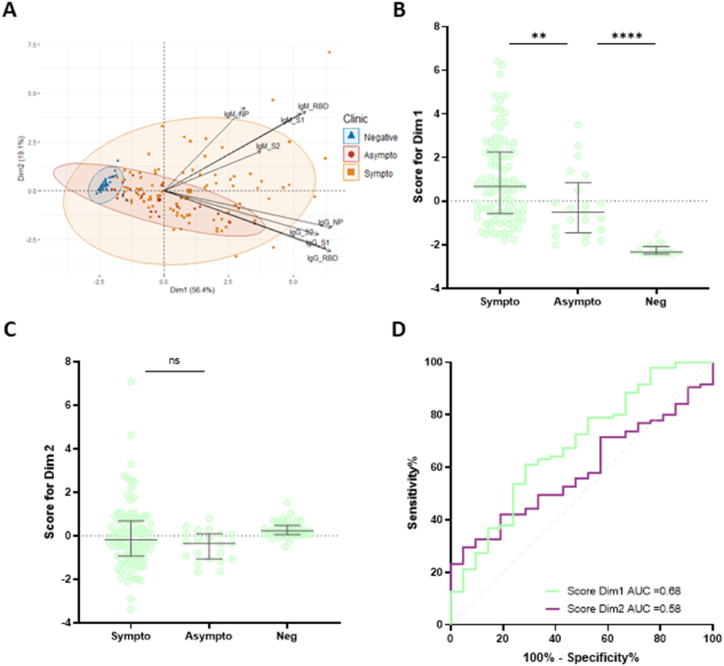
**PCA representing clinical presentation using serology results.** Principal component analysis (PCA) was performed on the expression of *anti*-S1, *anti*-S2, *anti*-RBD and *anti*-N IgM and IgG. A. Explanation of variance between among Symptomatic (N = 95) and asymptomatic (N = 21) patients all timepoints combined. Each point represents one patient. Color coding represents infection groups. The axes represent principal components 1 (Dimension 1, Dim 1) and 2 (Dimension 2, Dim 2) and the percentages indicate their contribution to the total observed variance. Axis values represent individual PCA scores. The concentration ellipses correspond to 90% data coverage. Arrows represent the contribution of each IgG and IgM to the variance described by Dim 1 and Dim 2. B. Distribution of individual PCA score values as a function of symptom presentation groups and time point, for Dim 1. Data were compared using the Mann-Whitney test. **: *p-value* <0.01 ****: *p-value* <0.0001 for comparisons between negative and 12 month. C. Distribution of individual PCA score values as a function of symptom presentation groups and time point, for Dim 2. Data were compared using the Mann-Whitney test. **: *p-value* <0.01 ****: *p-value* <0.0001 for comparisons between negative and 12 month. D. Receiving Operating Characteristic (ROC) curve obtained PCA Scores for Dim 1 and Dim 2.

Analysis of symptom presentation was performed using the scores obtained for Dim 1 and Dim 2 (Fig. 7A–D). There was a significant decrease in the Dim 1 score for the asymptomatic group compared to the symptomatic group (p = 0.0088) (Fig. 7B). These results were confirmed when the Dim1 score was added to the Dim 2 score. There was no difference between these two groups on Dim 2 (Fig. 7C).

ROC curves were performed to identify the discrimination between the symptomatic and the asymptomatic groups. This test revealed that the best discrimination between the two groups was obtained with the addition of Dim1 and Dim2 values (AUC = 0.71, 95% CI = 0.59–82; Specificity = 95.24% and sensitivity = 36.84%) (Fig. 7D and Table 3).

**Table 3:** Symptom presentation discrimination using PCA dimensions as scores.

	AUC	95% CI	p value	cut off	Sensitivity %	95% CI	Specificity %	95% CI
Dim1	0.68	0.55–0.81	0.0093	>2.56	21.05	14.06–30.29	95.24	77.33–99.76
Dim2	0.59	0.47–0.70	0.2215	>0.46	29.47	21.25–39.29	95.24	77.33–99.76
Dim1 + Dim2	0.71	0.59–0.82	0.0033	>0.93	36.84	27.83–46.88	95.24	77.33–99.76

### 3.5 Antibody responses describe time since infection and symptom presentation

A random forest classification model was used to estimate the date since SARS-CoV-2 infection and symptom presentation based on antibody expression. After removing the late converters (N = 7), 116 samples from D21 to 1-year post-infection and 40 negative samples, were analyzed. The model was trained on 126 samples (80% of the sample size). The model estimated 100 (32/32), 44.83% (16/29), 77.58% (45/58), and 0% were uninfected, infected for less than 3 months, 3–6 months, and 1 year, respectively (Table 4). For assessment of symptom presentation, the model predicted 93.42% (71/76) of symptom presentations but could not predict asymptomatic presentations (only 5.88% (1/17) of asymptomatic presentations were predicted) (Table 5).

**Table 4:** Estimation of the date of infection on the training set. % (N).

		Reference			
		Negative	<3 month	3–6 month	1 year
Prediction	**Negative**	**100** (32/32)	0	0	0
	**< 3 month**	0	**44.83** (16/29)	55,17 (13/29)	0
	**3**–**6 month**	0	17.24 (10/58)	**77.58** (45/58)	5.17 (3/58)
	**1 year**	14.29 (1/7)	0	85.71 (6/7)	**0**

**Table 5:** Prediction of symptom presentation on the training set. % (N).

		Reference		
		Negative	Asympto	Sympto
**Prediction**	**Negative**	**100** (32/32)	0	0
	**Asympto**	0	**5.88 (1/17)**	94.12 (16/17)
	**Sympto**	0	6.58 (5/76)	**93.42** (71/76)

The model was validated on the 30 remaining samples (20% of the sample size). The prediction accuracy was 80% (95% CI = 61.43–92.29, *p-value* = 0.0001) for the estimation of time since infection and 87.1% (95% CI = 70.17–96.37, *p-value* = 0.0016) for symptom presentation.

## 4 Discussion

Accurate SARS-CoV-2 seroprevalence data are key to better understand the burden of the SARS-CoV-2. Using the seroprevalence to further define time since infection and clinical presentation can be an important tool to better address and improve the response against COVID-19 especially in countries with poor diagnostic capacity.

In the present study we developed a multiplex assay to detect human *anti*-S1, *anti*-S2, *anti*-RBD and *anti*-N IgM and IgG antibodies. With the developed assay, we were able to observe the kinetics of antibody responses in patients with SARS-CoV-2 infection from day 1–12 months.

The first validation step allowed us to determine the performance of the test by measuring the sensitivity and specificity values. It is known that the N protein is well conserved within the coronavirus family (96% amino acid homology with SARS-Cov-1). Even if we obtained a fair sensitivity of 42%, we wanted to be as specific as possible for this assay even with IgM. Moreover, when testing a pool of pre-epidemic negative samples (2015), we found 2 patients strongly expressing *anti*-S2 IgG (MFI >10,000). These individuals were also positive for IgGs *anti*-S2 subunits of seasonal coronaviruses, suggesting high S2 cross-reactivity (Supplementary Fig. 2). It is also known that there is an active circulation of human coronaviruses (OC43 = 7.1%, NL63 = 3.7, 229 E = 0.7% HKU1 = 1%) in Madagascar [19,20].

Once the in-house Luminex xMAP® assay was validated, we compared the results obtained with 2 commercial ELISAs. Among all sera tested, 42/258 were considered negative with the Wantai kit and 78/258 with the ID. Vet kit. These negative samples were then tested with the Luminex xMAP® and respectively 23/42 and 31/78 were found positive using our in-house assay. These results confirmed that the Luminex xMAP® assay is more sensitive than the Wantai which is itself more sensitive than ID. Vet kit. Indeed, it has been described several times that Luminex xMAP® assay is more sensitive than conventional ELISA [[24], [25], [26]].

In the present study cohort, SARS-CoV-2 specific IgM and IgG were measured in symptomatic and asymptomatic COVID-19 confirmed patients. We noted no significant difference in antibodies to each of the targets, with the exception of anti RBD IgM. In general, it appears that IgM antibodies were higher in the symptomatic group than in the asymptomatic group. For IgG detection, the opposite trend was observed, with asymptomatic antibody levels being higher than those in the symptomatic group. These results have been described in several cohorts, with significant differences between these groups [12,14,27]. In the present cohort, only 8 patients were asymptomatic. The non-significant difference observed could be explained by the sample size.

We used this test to study the kinetics of seroconversion in patients over 1 year. IgG antibodies of anti S1, S2, RBD and N persisted overtime. During follow-up, we noticed that for 3 of the patients, antibodies seroconversion occurred only at 6 months. IgG seroconversion usually occur around the fifth and seventh day of symptom onset [28,29]. This seemingly surprising results could be explained by the fact that for these patients, either an infection by SARS-CoV-2 virus occurred between M3 and M6 after a possibly false-positive PCR test result was given at day 0, or a SARS-CoV-2 reinfection occurred after an initial infection that did not lead to seroconversion, or for these individuals’ seroconversion occurred almost 6 months after the initial infection. Indeed, the PCR test can have false-positive PCR results; one false-positive PCR can yield 6.3% meaning that these COVID-19 patients may have an initially false-positive RT-PCR result [30].

To visualize of the serological data, we performed a principal component analysis. Using dimensions 1 or 2 of the PCA as scores, ROC curves derived from these scores on dimension 1 showed an AUC of 0.98 for classification between infections from 3 months to 1 year and an AUC of 0.88 from 6 months to 1 year. The antibodies that contribute the most in the first dimension 1 are the *anti*-RBD IgGs. These results show that the decrease in MFI but persistence of seropositivity of *anti*-RBD IgGs over time seems to be a good marker for the discrimination of infections older than 1 year. Indeed, we found a persistence of *anti*-RBD IgG of more than 90% after one year of infection (Fig. 5). Dim 1 score of the PCA showed a significant decrease in the asymptomatic group compared to the symptomatic but no discrimination of symptomatic presentations versus asymptomatic ones could be achieved using the Dim2 score. Indeed, a subgroup of symptomatic seems to be segregated (high Dim2 score) from a larger mixed presentation group (low Dim2 score). Obtained scores showed a significant decrease of the asymptomatic one compared to symptomatic ones.

The random forest model made a few misclassifications within groups, however, the accuracy rate was >80% for predicting the time of infection and predicting symptom presentation. Symptomatic patients were classified better than non-symptomatic ones suggesting the existence of 2 biological groups of individuals: either symptomatic (IgM^high^) or a group of mixed presentations. This however may be because there are more symptomatic patients than asymptomatic patients in our cohort or because symptom presentation may be a subjective feeling. Nevertheless, it seems that IgM-producing individuals always feel symptomatic.

This study has several limitations, including sample size. This study was conducted on 43 patients who were followed for up to one year. But of these patients, only 13 completed the 12-month visit. The intervals of the group we chose for the estimation of time after infection could not give the best results. This observation is related to the fact that the number of patients in each interval class was too small, resulting in the misclassification of 3 months in the 6-months group and infections >3 months and 1 year in the 3 to 6-months group, which contains patients of 6 months and 3 months at the same time. We believe that these data need to be validated with a larger cohort.

A great asset of this study is the approach of using principal component analysis and a regression and classification model to retrospectively determine the time to infection and clinical symptoms presentation from a cohort of patients followed for one year and potentially over a year. Our group and others have shown that exposure to emerging VOCs can impact both the T-cell and the B-cell repertoires [31,32]. Indeed, in Madagascar, it was shown that during the second epidemic wave in 2021, antibody affinity gradually shifted towards VOC^Beta^. These multiple antigen affinities and their evolution following successive immunisations, whether they be vaccinal or “natural”, could be integrated in a more complex model and thoroughly describe a history of populational exposure to SARS-CoV-2.

The results of this study can be used for surveillance of a population where exposure to SARS-CoV-2 is poorly established. This innovative approach could help to investigate spatio-temporal dynamics and epidemiological surveillance of other infectious diseases. Since this study was performed before the vaccination campaign in Madagascar, it is only relevant in an unvaccinated population study. Adjustments would thus be necessary to evaluate natural infection serological responses in a vaccinated population. Moreover, this serological infection timing tool could be used in cohorts of individuals exposed to other pathogens and comorbidities associated to SARS-CoV-2, such as *Mycobacterium tuberculosis*, to study the impact of co-infections as risk factors provided the response to both pathogens does not impact the humoral response to either pathogens or its impact is integrated to the model. This may help evaluate the impact of the ongoing COVID-19 pandemic on other public health priorities such as tuberculosis, HIV, malaria or other respiratory diseases [[33], [34], [35], [36]].

## Fundings

The study was funded by the French Ministry for Europe and Foreign Affairs through the REPAIR COVID-19-Africa project coordinated by the Pasteur International Network association. WANTAI reagents were provided by 10.13039/100004423WHO AFRO as part of a Sero-epidemiological “Unity” Study Grant/Award Number: 2020/1019828-0 P.O 202546047 and Initiative 5% grant n°AP-5PC–2018–03-RO.

## Author contribution statement

Mame Diarra Bousso NDIAYE: Conceived and designed the experiments; Analyzed and interpreted the data; Wrote the paper.

Lova Tsikiniaina RASOLOHARIMANANA, Solohery Lalaina RAZAFIMAHATRATRA, Rila RATOVOSON, Voahangy RASOLOFO, Paulo RANAIVOMANANA, Laurent RASKINE, Jonathan HOFFMANN, Rindra RANDREMANANA and Niaina RAKOTOSAMIMANANA: Contributed reagents, materials, analysis tools or data.

Lova Tsikiniaina RASOLOHARIMANANA: Performed the experiments; Analyzed and interpreted the data.

Matthieu SCHOENHALS: Conceived and designed the experiments; Performed the experiments; Analyzed and interpreted the data; Contributed reagents, materials, analysis tools or data; Wrote the paper.

## Data availability statement

Data will be made available on request.

## Declaration of competing interest

The authors declare that they have no known competing financial interests or personal relationships that could have appeared to influence the work reported in this paper.
