# Supplementary material for: Using a multiplex serological assay to estimate time since SARS-CoV-2 infection and past clinical presentation in malagasy patients
Source: Heliyon. 2023 Jun 13;9(6):e17264. doi: 10.1016/j.heliyon.2023.e17264 (PMC10263216; doi:10.1016/j.heliyon.2023.e17264)
Supplement: Multimedia component 1 [file mmc1.docx]

**List of supplementary tables:**

**Supplementary Table 1**: Characteristics of the cohorts

**Supplementary Table 2**: Validation of the Luminex assay

**Supplementary Table 3:** Antibodies and recombinant proteins.

**Supplementary Table 4**: Percentage of IgM and IgG seroconversion.

**Supplementary Table 1:** Characteristics of the cohorts

| **Patients of the study** | Evaluation |
| --- | --- |
| Sample collection date | March 2020 - July 2020 |
| Number included | 43 |
| Number of samples | 271 |
| Gender |  |
| Male | 42% (18/43) |
| Female | 58% (25/43) |
| Age (years) | 43 (15-71) |
| **Symptoms** |  |
| Symptomatic | 81% (35/43) |
| Non symptomatic | 19% (8/43) |
| **Comorbidities** |  |
| Diabetes | 7% (3/43) |
| Asthma | 2.3% (1/43) |
| Obesity | 2.3% (1/43) |
| Cardiovascular disease | 7% (3/43) |
|  |  |
| **Negative controls** |  |
| Collection date | 2015 |
| Number included | 40 |

**Supplementary Table 2: Validation luminex assay.** Characteristics of validation for the luminex assay. LLoQ: Lower Limit Of Quantification. ULoQ: Upper Limit Of Quantification. CV intra essai < 10%CV inter essai <15%.

|  | **Spike S1** | **Spike S2** | **Spike RBD** | **N** |
| --- | --- | --- | --- | --- |
| **CV% intra assay** | 2.87 | 2.14 | 2.53 | 2.21 |
| **CV% inter assay** | 3.08 | 3.12 | 2.96 | 3.38 |
| **LLoQ Ab (ng/mL)** | 0.1 | 0.1 | 0.1 | 0.1 |
| **ULoQ Ab (ng/mL)** | 1000 | 1000 | 1000 | 1000 |

**Supplementary Table 3**: Antibodies and recombinant proteins: Ab: Antibody. Detection antibodies are coupled with Phycoerythrine (PE).

| **Analytes** | **Reagent** | **Reference** | **Supplier** |
| --- | --- | --- | --- |
| **SARS-CoV-2 Spike S1** | Recombinant protein | Z03485 | GenScript |
|  | Capture Ab | A02038-100 |  |
| **SARS-CoV-2 Spike S2** | Recombinant protein | 40590-V08B | Sinobiological |
|  | Capture Ab | 40590-D001 |  |
| **SARS-CoV-2 RBD** | Recombinant protein | Z03483 | GenScript |
|  | Capture Ab | A02046-100 |  |
| **SARS-CoV-2 N** | Recombinant protein | Z03480 | GenScript |
|  | Capture Ab | A02039-100 |  |
| **HCoV-HKU1 Spike S1** | Recombinant protein | 40021-V08H | Sinobiological |
| **HCoV-NL63 Spike S1** | Recombinant protein | 40600-V08H |  |
| **HCoV-OC43 Spike S1** | Recombinant protein | 40607-V08H1 |  |
| **HCoV-229E Spike S1** | Recombinant protein | 40601-V08H |  |
| **Anti-human IgG PE** | Detection Ab | H10104 | Lifetechnologies |
| **Anti-human IgM PE** | Detection Ab | H15104 |  |

**Supplementary Table 4:** Percentage of IgM and IgG seroconversion

|  |  | **IgM** | | | |  | **IgG** | | | |
| --- | --- | --- | --- | --- | --- | --- | --- | --- | --- | --- |
| **Days** |  | **Spike S1** | **Spike S2** | **RBD** | **N** |  | **Spike S1** | **Spike S2** | **RBD** | **N** |
| **1** |  | 81.40 | 41.86 | 55.81 | 30.23 |  | 65.12 | 62.79 | 69.77 | 72.09 |
| **7** |  | 93.02 | 60.47 | 65.12 | 44.19 |  | 86.05 | 86.05 | 86.05 | 83.72 |
| **14** |  | 93.02 | 65.12 | 65.12 | 46.51 |  | 86.05 | 86.05 | 86.05 | 83.72 |
| **21** |  | 93.02 | 65.12 | 67.44 | 46.51 |  | 86.05 | 86.05 | 86.05 | 83.72 |
| **90** |  | 95.35 | 67.44 | 69.77 | 46.51 |  | 88.37 | 88.37 | 88.37 | 86.05 |
| **180** |  | 100 | 69.77 | 72.09 | 48.84 |  | 90.70 | 93.02 | 93.02 | 90.70 |
| **365** |  | 100 | 75.81 | 72.09 | 70.76 |  | 95.35 | 100 | 100 | 95.35 |
