# Supplementary material for: Using a multiplex serological assay to estimate time since SARS-CoV-2 infection and past clinical presentation in malagasy patients
Source: Heliyon. 2023 Jun 13;9(6):e17264. doi: 10.1016/j.heliyon.2023.e17264 (PMC10263216; doi:10.1016/j.heliyon.2023.e17264)
Supplement: Multimedia component 2 [file mmc2.docx]

**List of supplementary figures:**

**Supplementary Figure 1: Validation luminex assay**. Coupling confirmation and limits of detection of anti–SARS-CoV-2 Spike S1, nucleocapsid N protein IgG, anti–SARS-CoV-2 Spike S1 IgG and anti–SARS-CoV-2 Spike RBD IgM antibodies by luminex. (A) Confirmation of the coupling step. A plateau of MFI was reached for dilutions 2μg/mL. (B) A calibration curve was obtained after serial dilutions of capture antibodies specific of each targeted protein. Lower and upper limit of quantifications are represented by dashed grey lines. PE: Phycoerithryn; MFI: Median of Fluorescence Intensity; Ab: Antibody.

**Supplementary Figure 2: cross-reactivity with HCoV**. Red dots pre-epidemic patients who cross-react with seasonal coronavirus.

**Supplementary Figure 3: Comparison of the in-house luminex assay and 2 ELISA commercial assay**. Patients with negative ELISA ID.vet (n=78) and Wantai (n=42) results. All timepoints combined were surveyed for SARS-CoV-2 antibodies using the luminex assay. (A) Represents IDVET tested IgM and IgG. (B) Represents Wantai tested IgM and IgG. Pre_epidemic samples were used to set the cut off wich is indicated with dashed line and orange area. Each dot represent a sample. Red squares represent targets of Elisa tests

**Supplementary Figure 4: Percentage of explained variances of each dimension of the PCA**. Dim1 and Dim2, used to represent the PCA graphs, together explain 75.5% of the variances. Numbers are eigenvalues in percent.


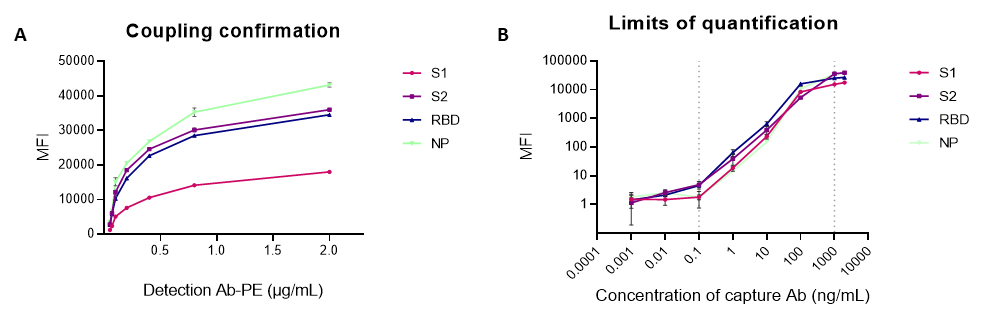


**Supplementary Figure 1 : Validation luminex assay**. Coupling confirmation and limits of detection of anti–SARS-CoV-2 Spike S1, nucleocapsid N protein IgG, anti–SARS-CoV-2 Spike S1 IgG and anti–SARS-CoV-2 Spike RBD IgM antibodies by luminex. (A) Confirmation of the coupling step. A plateau of MFI was reached for dilutions 2µg/mL. (B) A calibration curve was obtained after serial dilutions of capture antibodies specific of each targeted protein. Lower and upper limit of quantifications are represented by dashed grey lines. PE: Phycoerithryn; MFI: Median of Fluorescence Intensity; Ab: Antibody.

**Supplementary Figure 2: Cross-reactivity with HCoV**. The negative pre-epidemic sera were tested for seasonal coronaviruses. Red dots represent patients who cross-react with specific seasonal coronavirus antigens.


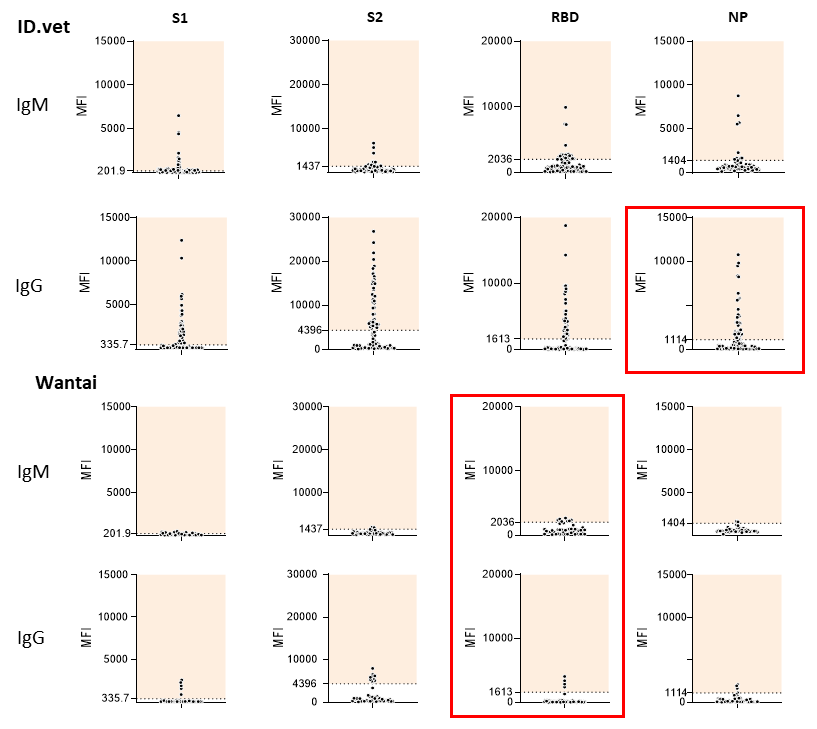


**Supplementary Figure 3: Comparison of the in-house luminex assay and 2 ELISA commercial assay**. Patients with negative ELISA ID.vet (n=78) and Wantai (n=42) results. all timepoints combined were surveyed for SARS-CoV-2 antibodies using the luminex assay. (A) represents IDVET tested IgM and IgG. (B) represents Wantai tested IgM and IgG. Pre_epidemic samples were used to set the cut off wich is indicated with dashed line and orange area. Each dot represent a sample. Red squares represent targets of Elisa tests.


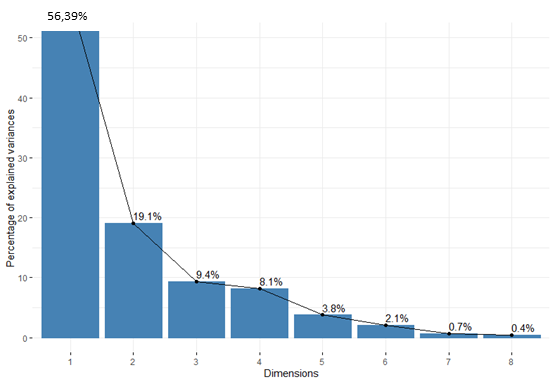
**Supplementary Figure 4: Percentage of explained variances of each dimension of the PCA**. Dim1 and Dim2, used to represent the PCA graphs, together explain 75.5% of the variances. Numbers are eigenvalues in percent.
